# Public health round-up

**DOI:** 10.2471/BLT.24.010424

**Published:** 2024-04-01

**Gaza crisis deepens**

United Nations field operatives supplying a hospital in the Gaza Strip where malnutrition is adding to the challenges faced by the population. According to the World Health Organization’s (WHO) latest Operational Update on Health Emergencies released on 7 March, the ongoing hostilities and restricted humanitarian access to essential resources pose a serious risk of famine in the coming months.

**Figure:**
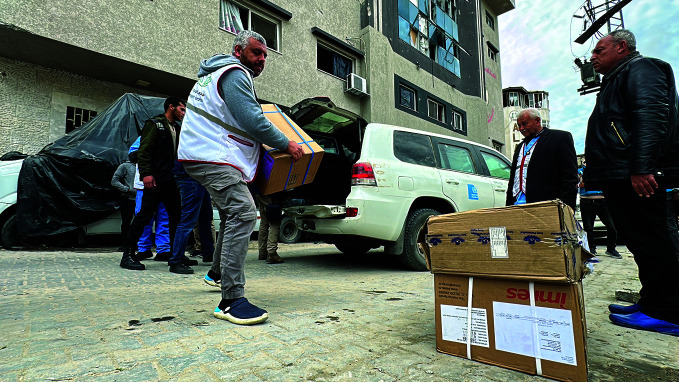
Courtesy of WHO

## Risk of famine in Gaza

Six months into the hostilities in the Gaza Strip, the health crisis is impacting the majority of the roughly two million residents, 75% of whom have been displaced. The population faces challenges that include the disruption of essential services, significant trauma and – increasingly – malnutrition and infectious diseases.

A January 2024 nutrition vulnerability and situation analysis report revealed a rapid deterioration in nutritional status, with acute malnutrition rates exceeding 15% among children aged 6–23 months. Nearly 3% of these children suffer from severe wasting.

The latest integrated food security and nutrition phase classification (IPC) report covering the period between 8 December 2023 and 7 February 2024 classified the entire Gaza Strip population as IPC Phase 3 or above (crisis or worse), the highest proportion ever classified by the IPC initiative for acute food insecurity.

In a 21 February statement, Principals of the Inter-Agency Standing Committee called on Israel to fulfil its legal obligation under international humanitarian and human rights law to provide food and medical supplies and facilitate aid operations in Gaza.

Despite difficult conditions, WHO and partners were able to reach Al-Shifa hospital and Al-Helou hospital on 11 March to deliver fuel, medical supplies and food, according to an 11 March X post by World Health Organization (WHO) Director-General Tedros Adhanom Ghebreyesus.

https://bit.ly/3ViFAWf

https://bit.ly/3uZojqz

https://bit.ly/3Ix1jST

https://bit.ly/3wSL0NF

## HIV resistance concerns

Observational data indicate that levels of human immunodeficiency virus (HIV) resistance to dolutegravir-containing antiretroviral therapy is exceeding levels observed in clinical trials.

WHO has recommended use of dolutegravir as the preferred first- and second-line HIV treatment for all population groups since 2018. It is more effective, easier to take, and has fewer side-effects than other drugs currently in use.

However, as reported in WHO’s HIV Drug Resistance Report, released on 5 March, resistance to dolutegravir has been reported as reaching 19.6% among people experienced with treatment and transitioning to a dolutegravir-containing antiretroviral therapy while having high HIV viral loads.

“The worrying evidence of resistance in individuals with unsuppressed viral load despite dolutegravir treatment underscores the necessity for increased vigilance and intensified efforts to optimize the quality of HIV care delivery,” said Dr Meg Doherty, Director, WHO Department of the Global HIV, Hepatitis and STI Programmes.

https://bit.ly/3TfFsUJ

## Under-5 mortality falls

The number of children dying before their fifth birthday has reached a historic low, according to the report released on 13 March by the United Nations Inter-agency Group for Child Mortality Estimation.

The report reveals that global under-5 mortality has fallen by 51% from an estimated 9.9 million in 2000 to an estimated 4.9 million in 2022. Several low- and lower-middle-income countries showed that mortality reductions of 75% are possible when resources are sufficiently allocated to primary health care.

Despite this progress, 4.9 million children died before the age of 5 – nearly half of whom were newborns –prompting Li Junhua, United Nations Under-Secretary-General for Economic and Social Affairs to say that further efforts and investments are needed to reduce inequities and end preventable deaths.

https://bit.ly/4ca7AkR

## The growing obesity burden

The total number of children, adolescents and adults worldwide living with obesity has surpassed one billion. This is one of the main findings of a study released by The Lancet on 29 February estimating the combined and individual prevalence of underweight and obesity, and their changes, from 1990 to 2022 for adults and school-aged children and adolescents in 200 countries and territories.

The study reveals that the number of women with obesity reached 504 million in 2022, up from 127 million in 1990, an increase of 377 million. The number of men with obesity reached 374 million in 2022, up from 67 million in 1990, an increase of 307 million. An additional 159 million children and adolescents were also living with obesity in 2022. The countries with the largest absolute numbers of adults with obesity in 2022 were the United States of America, China and India.

The study also shows that even though the rates of undernutrition have dropped, it is still a public health challenge in many places, particularly in the South-east Asian and African regions.

https://bit.ly/3IACnd2

https://bit.ly/4adSSHZ

## Tackling cervical cancer

Governments, donors, multilateral institutions and partners announced major new policy, programmatic and financial commitments to eliminating cervical cancer.

Announced at the first-ever global cervical cancer elimination forum which took place in Cartagena de Indias, Colombia, between 5 to 7 March, the commitments include nearly US$ 600 million in new funding, and country-level plans to expand vaccine coverage and strengthen screening and treatment programmes.

https://bit.ly/49M4mSZ

## Pathogen genome surveillance

WHO announced US$ 4 million in funding from donors to create a catalytic grant fund for organizations working in pathogen genomic surveillance.

The initial grants for the catalytic fund have been provided by the Bill & Melinda Gates Foundation, the Rockefeller Foundation and Wellcome, to support the international pathogen surveillance network, a new global network of pathogen surveillance actors, convened by WHO. The fund’s purpose is to empower lower-resource members of the network to create knowledge that can benefit the global genomics surveillance community.

https://bit.ly/48LO0bX

## Gender inequality in health and care work

Gender inequality in health and care work negatively impact women, health systems and health outcomes. This is among the key findings of *Fair share for health and care: gender and the undervaluation of health and care work*, a report published by WHO on 13 March.

The report sets out the ways in which underinvestment in health systems results in a vicious cycle of unpaid health and care work, lowering women’s participation in paid labour markets, harming women’s economic empowerment and hampering gender equality.

https://bit.ly/48QzmAc

**Cover photo**

A young woman and her mother-in-law sit at their home in rural Nepal.

**Figure:**
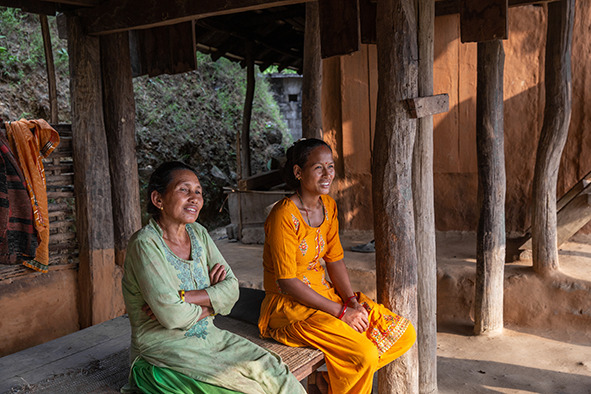
WHO/HRP/Uma Bista

## Hearing aid guidance

WHO released new technical guidance on hearing aid service delivery approaches for low- and middle-income settings. The guidance makes practical recommendations on how to develop hearing aid services in countries that lack human resources for assessing hearing, as well as fitting and maintaining hearing aids.

Developed with support from the ATScale Global Partnership for Assistive Technology, the guidance is based on the principle of task-sharing among specialists and trained non-specialists, and focuses on targeting adults and children aged five years and older. It also includes resources relating to healthy ear care practices, use of hearing aids and how to support people living with hearing loss.

https://bit.ly/3IFfuoI

## New mental health guidance

WHO published a new, comprehensive diagnostic manual for mental, behavioural, and neurodevelopmental disorders reflecting updates to the *International statistical classification of diseases and related health problems, 11th revision* (ICD-11). It includes guidance on diagnosis for several new categories added in ICD-11, including complex post-traumatic stress disorder, gaming disorder and prolonged grief disorder.

Published on 8 March, the manual is designed to support qualified mental health and other health professionals to identify and diagnose mental, behavioural and neurodevelopmental disorders in clinical settings.

In related news, WHO released a new manual to facilitate the application of psychological interventions, aiming to narrow the treatment gap in mental health care.

Released on 11 March, the manual emphasizes evidence-based psychological interventions administered by non-specialists, offering practical instructions for their implementation. These interventions, derived from established treatments like cognitive behavioural therapy and stress management, have shown effectiveness, particularly in addressing depression and anxiety.

Globally, mental health conditions affect 1 in 8 individuals. However, the majority remain untreated due to various barriers like inadequate services, stigma, and affordability issues.

https://bit.ly/3PjypJQ

https://bit.ly/3PlF4Tz

**Looking ahead**

17–18 April. Sixth Global Ministerial Summit on Patient Safety 2024. Santiago, Chile. https://bit.ly/3VfCoeb

24–30 April. World Immunization Week 2024. https://bit.ly/3TiFviy

26 April. High-level meeting to defeat meningitis. Institut Pasteur Paris, France. https://bit.ly/3VgUgVV

